# Single-cell proteomic analysis reveals Multiple Myeloma heterogeneity and the dynamics of the tumor immune microenvironment in precursor and advanced states

**DOI:** 10.1016/j.neo.2025.101189

**Published:** 2025-06-06

**Authors:** Mohamed Kamal, Stephanie N. Shishido, Jeremy Mason, Krina Patel, Elisabet E. Manasanch, Robert Z. Orlowski, Peter Kuhn

**Affiliations:** a Convergent Science Institute for Cancer, Michelson Center, University of Southern California, Los Ange-les CA 90089, USA; b Department of Lymphoma and Myeloma, USA; c Department of Experimental Therapeutics, Division of Cancer Medicine, University of Texas MD Anderson Cancer Center, Houston, TX, 77030, USA; d Catherine & Joseph Aresty Department of Urology, Institute of Urology, Keck School of Medicine, University of Southern California, Los Angeles, CA 90033, USA; e Norris Comprehensive Cancer Center, Keck School of Medicine, University of Southern California, Los Angeles CA 90033, USA; f Department of Biomedical Engineering, Viterbi School of Engineering, University of Southern California, Los Angeles, CA 90089, USA; g Department of Aerospace and Mechanical Engineering, Viterbi School of Engineering, University of Southern California, Los Angeles, CA 90089, USA; h Department of Biological Sciences, Dornsife College of Letters, Arts, and Sciences, University of Southern California, Los Angeles, CA 90089, USA

**Keywords:** Multiple myeloma, Plasma cells, Tumor immune microenvironment, Proteomics

## Abstract

Multiple myeloma (MM) is an aggressive hematologic malignancy arising from plasma cell (PC) proliferation in the bone marrow, progressing from its precursor states MGUS and SMM. Despite therapeutic advances, MM remains incurable, underscoring the need for better risk stratification and early detection. Tumor heterogeneity and dynamic immune microenvironment changes drive progression, yet bulk analyses overlook rare subpopulations critical to disease evolution and resistance. This study employed multiplexed targeted proteomics to characterize bone marrow aspirates (BMA) from 22 patients to observe the change in the distribution of PCs and tumor immune microenvironment (TiME) cells across MM disease states and controls. Bone marrow samples were processed, stained with a 29-metal-labeled antibody panel, and analyzed using computational clustering approaches. Clustering of PCs revealed changes in subtypes with disease progression, marked by shifts from CD45-positive/CD138-low subpopulations in precursor states to CD45-negative/CD138-high populations in advanced MM. Analysis of the TiME identified distinct immune phenotypes, with significant reductions in monocyte/macrophage and lymphoid clusters across MM states compared to controls. Notably, a distinct PC cluster enriched in NDMM and RRMM exhibited high BCMA and CD138 expression, suggesting a potential role in disease progression. These findings provide critical insights into MM evolution and immune landscape alterations, with implications for targeted therapeutic strategies.

## Highlights

• Multiplexed proteomics reveals MM progression-associated PC and immune shifts.
• CD45-negative/CD138-high PC populations expand in advanced multiple myeloma.
• Distinct immune phenotypes emerge with MM progression, reducing monocyte/macrophage cells.
• A BCMA/CD138-high PC cluster is enriched in NDMM and RRMM, indicating disease impact.
• Findings enhance MM risk stratification and inform targeted therapeutic strategies.

## Introduction

Multiple myeloma (MM) is an aggressive hematologic malignancy characterized by the clonal proliferation of plasma cells (PCs) in the bone marrow, with patients commonly suffering from bone lesions, renal insufficiency, hypercalcemia, and bone marrow failure [1,2]. MM is the second most common hematologic cancer, and its global incidence has surged by 126 % over the past three decades, with mortality increasing by 94 % [3]. Despite significant therapeutic advances, MM remains incurable, highlighting the urgent need for early detection, improved risk stratification, and novel therapeutic strategies for improving patient outcomes.

MM evolves from precursor conditions such as monoclonal gammopathy of undetermined significance (MGUS) and smoldering multiple myeloma (SMM). These precursor states are characterized by an abnormal expansion of PCs in the bone marrow, eventually progressing to symptomatic MM, relapsed/refractory MM (RRMM), and, in some cases, primary or secondary plasma cell leukemia (PCL). MGUS is defined by the presence of fewer than 10 % monoclonal PCs in the bone marrow without associated symptoms [4]. While some MGUS patients remain stable for decades, the annual risk of progression to MM is ∼1 % per year, whereas SMM progresses at a rate of ∼10 % per year over the first five years, influenced by underlying cytogenetic abnormalities [5]. But many patients with MGUS or SMM will never progress to symptomatic disease. Given the unpredictable nature of clonal plasma cell dyscrasias, developing reliable tools for risk stratification and early detection of precursor states is crucial. This is particularly relevant for SMM, where recent clinical trials could lead to FDA-approved treatments that redefine standard of care [6]. Given the potential high cost and relatively small population that ultimately will benefit from interventions for SMM, it is critical to stratify those high-risk individuals who could benefit from early therapeutic intervention while minimizing unnecessary intervention for those with a low likelihood of progression. Managing ‘at-risk’ populations with precursor conditions is a major challenge in both hematologic and solid cancers and addressing this challenge could improve patient care and resource allocation.

Development and progression of MM is a highly heterogeneous and dynamic process, making it difficult to accurately assess asymptomatic patients, which has traditionally led to a ‘wait and see’ approach in clinical practice. Most MM studies have relied on bulk tissue analyses, which lack the resolution to identify rare tumorigenic subpopulations that drive disease progression from MGUS to overt disease and residual disease relapse. Recent single-cell studies have highlighted the subclonal and longitudinal evolution of MM [7], emphasizing the need for a deeper understanding of its heterogeneity. Further, the tumor immune microenvironment (TiME) plays a critical role in MM pathophysiology, disease progression, and drug resistance [8,9]. However, due to the inherent heterogeneity and evolving TiME, no single protein biomarker can definitively diagnose the disease. The evolving proteomic landscape across cell types throughout MM progression and relapse remains underexplored, yet it holds potential for identifying diagnostic and prognostic biomarkers. Proteomics has emerged as a powerful tool in cancer research, enabling the discovery of new therapeutic targets and the development of predictive models to monitor disease progression [10]. Highly multiplexed proteomic techniques such as PhenoCycler, multiplexed ion beam imaging by time of flight (MIBI-TOF), and imaging mass cytometry (IMC) allow for high-resolution spatial analyses of MM and its microenvironment without disrupting sample integrity [11,12], providing new opportunities to examine the MM PCs and the interactions with the TiME.

**Fig. 1:**
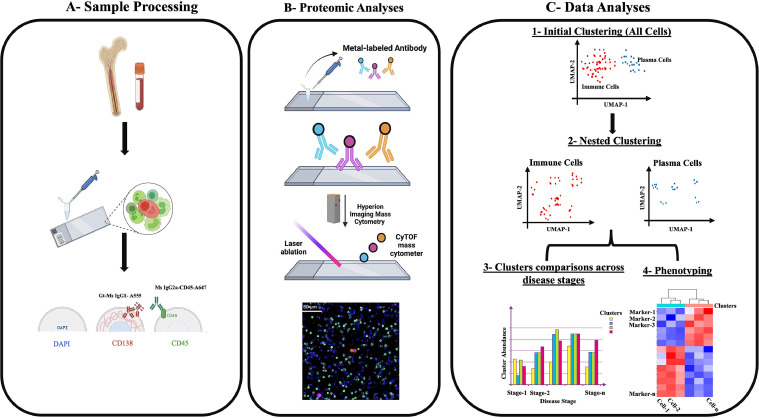
**HDSCA-IMC workflow.** A. BMA samples are received, undergo processing by red blood cell lysis, are plated onto custom glass slides at approximately 3 million cells per slide and are then stained with our 3-color immunofluorescence assay. B Slides are stained with a panel of metal-labeled antibodies, laser ablated using the Hyperion IMC system, and multiplexed images are generated. C. IMC data have been used to perform a nested clustering to detect PCs and TiME subtypes. Abundance of different subtypes have been compared across disease states and phenotyped using the top differentially expressed markers in each subtype compared to control.

Understanding how distinct subtypes of PCs and TiME cells contribute to disease progression is critical for improving diagnosis and treatment strategies. The goal of this study was to identify prevalent subtypes of PCs and TiME cells in the bone marrow aspirate (BMA) relevant to MM and investigate whether these subpopulations change across different MM disease states. To achieve this, we utilized the high-definition single-cell assay (HDSCA) and imaging mass cytometry (IMC) to analyze the expression of 29 protein markers at the single-cell level in a subset of patients and controls (Fig. 1). A key question underlying our approach was determining the number of cells required to define a cluster, given the dynamic nature of cellular composition. We hypothesized that the distribution of PC and TiME subpopulations shifts with disease progression, reflecting underlying biological changes in MM. Our findings provide a high-resolution view of MM cellularity, offering new insights into disease biology that may inform diagnostic, prognostic, and therapeutic strategies.

### Study design

The study was conducted with approval from the Institutional Review Board (IRB) in accordance with ethical principles founded in the Declaration of Helsinki, including the International Conference on Harmonization, Good Clinical Practice regulations and guidelines, and all applicable local regulations. Bone marrow aspirates (BMA) were collected from patients with different stages of MM (MGUS, SMM, NDMM, RRMM; 4 to 5 patients per stage). All MM patients were recruited at The University of Texas MD Anderson Cancer Center (Houston, TX; IRB: UP-19-0033). For diagnostic evaluation, all enrolled MM patients underwent bone marrow biopsy and serological testing. Each patient's corresponding sample was analyzed using standard-of-care flow cytometry at MD Anderson as part of the diagnostic assessment. Patient samples were collected between 04/2019-08/2021. BMA samples from 3 organ-confined prostate cancer patients served as controls and will be referred to as such throughout (IRB: HS-21-00292 and NA_00087094).

### Sample processing

BMA samples (8 mL each) were collected in EDTA tubes at MD Anderson and transported to the Convergent Science Institute in Cancer at the University of Southern California within 48 hours, following established protocols [[13], [14], [15]]. Upon arrival, samples were immediately subjected to red blood cell lysis using an isotonic ammonium chloride solution. The resulting nucleated cells were then plated as a monolayer onto custom-made cell adhesive glass slides (Marienfeld, Lauda, Germany). White blood cell (WBC) counts were utilized to estimate the plating of 3 million cells per slide. The prepared slides were subsequently processed for HDSCA [16] and IMC (Fig. 1).

### Multiplexed targeted proteomics

Multiplexed targeted proteomics analysis was conducted using Imaging Mass Cytometry (IMC) on the Standard Biotools Hyperion machine on a total of 22 patient samples. A median of 48.5 (range 20 to 52) regions of interest (ROIs), each measuring 400 µm², were randomly selected and analyzed per sample. Each ROI contained between 4 and 8745 (median 364) nucleated cells.

### Antibody staining

The slides were stained with a 29-metal-labeled antibody panel specific to MM and bone marrow microenvironment, along with 2 DNA intercalators (Table 1). Each antibody was optimized individually using U266 cells spiked into normal blood donor (NBD) samples.

**Table 1:** IMC multiplexed proteomic analysis panel of metal-labeled antibodies.

Target	Metal Tag	Antibody Clone
BCMA	150 Nd	Rabbit_IgG_EPRBOB-R1-F1-24
CCR4 (CD194)	175 Lu	Mouse_IgG2b_205410
CCR7	167 Er	Rabbit_IgG_Y59
CD3	170 Er	Rabbit_IgG_Polyclonal
CD4	176 Yb	Rabbit_IgG_EPR6855
CD8a	Dy162	Rabbit_IgG_D8A8Y
CD11c	159 Tb	Mouse IgG1 kappa BU15
CD14	156 Gd	Rabbbit_IgG_EPR3653
CD16	209 Bi	Mouse_IgG1k_3G8
CD25 (IL-2)	171 Yb	Rabbit_IgG_EPR6452
CD27	144 Nd	Rabbit_IgG_EPR8569
CD28	153 Eu	Rabbit_IgG/EPR22076
CD38	152 Sm	Rabbit IgG/epr22691-219
CD45	89 Y	Mouse_IgG1k_HI30
CD45RA	166 Er	Mouse_IgG2bk_HI100
CD45-RO	173 Yb	Mouse_IgG2a_UCHL1
CD56	149 Sm	Mouse_IgG2b k_NCAM16.2
CD62L	158 Gd	Rabbit_IgG_414
CD66b (CEACAM)	169 Tm	Rabbit_IgG_EPR20721
CD69	160 Gd	Rabbit_IgG_EPR21814
CD117	145 Nd	Rabbit_IgG_YR145
CD123	151 Eu	Mouse_IgG1_6H6
CD127	164 Dy	Rabbit_IgG_EPR2955(2)
CD138/ Syndecan	148 Nd	Rabbit IgG_EPR6454
CD161	168 Er	Mouse_IgG1_OTI1D8
CD223/LAG3	155 Gd	Rabbit_IgG_EPR20261
CXCR3 (CD183)	163 Dy	Mouse_IgG1_G025H7
HLA-DR	174 Yb	Mouse_IgG2ak_L243 (helios)
PD-1	165 Ho	Rabbit_IgG_CAL20

These targets are critical for characterizing the PC compartment and TiME. BCMA is essential for plasma cell survival and is targeted in multiple myeloma therapies, while CD138 (Syndecan-1) and CD38 are also key plasma cell markers. T-cell subsets are defined by CD3, CD4, CD8a, CD45RA, and CD45RO, with CD27 and CD28 providing costimulatory signals, and CD69 marking early activation. PD-1 and CD223 (LAG-3) are immune checkpoints involved in T-cell exhaustion, contributing to immune evasion. CCR4, CCR7, CXCR3 (CD183), and CD62L regulate immune cell trafficking, while CD127 (IL-7R alpha) supports T-cell survival. CD16 and CD56 play roles in NK cell-mediated cytotoxicity, and CD11c, CD14, CD66b (CEACAM), and HLA-DR mark antigen-presenting and myeloid cells involved in immune activation. CD25 (IL-2R alpha) and CD161 are important for regulatory and cytotoxic T-cell function. CD117 (c-Kit) and CD123 (IL-3R alpha) are involved in hematopoietic progenitor and dendritic cell biology. These markers help delineate immune cell populations, tumor interactions, and therapeutic targets, making them essential for understanding and manipulating the immune landscape in cancer.

### Image preprocessing

IMC data were collected to reconstruct 31-dimensional images (.mcd files) with a spatial resolution of ∼1 μm² across the ROI. MCD (.mcd) files generated from the Hyperion Imaging System (RRID: SCR_023195) were visualized using HistoCAT (version 2.2) and MCDViewer (version 1.0.560.6, RRID: SCR_023007) to assess staining quality. Cell boundary segmentation and pixel values were determined using a customized pipeline involving CellProfiler, ilastik, and histoCAT as previously reported [[16], [17], [18]]. Classification of nuclei, membranes, and background regions was performed using ilastik, with results exported as probability masks. Single-cell measurements were then extracted from all channels, employing the scikit-image package (version 0.14.5). The signal output was calculated as the mean ion counts per cell area, subtracting the background signal determined from negative mask space. Antibody quality was visually inspected using both MCD viewer (whole ROIs) and an in-house Python-based pipeline to visualize rarely expressed markers.

### Clustering

Single cell measurement data were cleaned to remove noise by applying thresholds on area (minimum 35 and maximum 650 pixels) and DNA (> 0.3). Prior to removing noise there were 326,186 potential events, post cleaning resulted in 289,289 cells. Data were preprocessed, reduced dimensionality, and clustered using the Seurat package in R (version 4.4.1). Data were normalized using Centered Log Ratio (CLR). Highly variable features were identified, and expression values were standardized, ensuring a mean of zero and a standard deviation of one. Principal Component Analysis (PCA) was performed on scaled data using five principal components. K-nearest-neighbors' graphs were constructed, and clustering was conducted using the Leiden or Louvain algorithm. Clusters were visualized using Uniform Manifold Approximation and Projection (UMAP), while marker expression across clusters was examined using a heatmap. Cell clusters exhibiting marker expression profiles resembling PCs were subset into a separate object, where PCA was performed on scaled data with 25 principal components. The subsequent clustering process followed the same steps as described above. **Supplemental Table 1** provides the cell count per cluster (Primary, PC, and TiME).

### Statistical analyses

Pairwise Wilcoxon tests were performed to compare the number of cells per cluster per 100,000 cells between the control group and other disease stages. P value < 0.05 was considered significant. Non-parametric statistical methods were applied to assess correlations between rare events identified in liquid biopsy samples and clinical parameters. The Wilcoxon rank sum test was used for categorical variables, while Spearman's rank correlation was employed for continuous and ordinal data.

## Results

### Patient information

This study includes 22 BMA samples from 19 patients across four MM disease states (MGUS, SMM, NDMM and RRMM) plus 3 prostate cancer cases that serve as controls. Patients’ clinical information is provided in Table 2.

**Table 2:** **Patient clinical information.** Follow-up time was calculated as the difference between the date of death or last contact date and the date of BM sampling.

**Variable**	**All (*n* = 19)**	**MGUS (*n* = 5)**	**SMM (*n* = 5)**	**NDMM (*n* = 5)**	**RRMM (*n* = 4)**
M-spike Value (SPEP**) (g/dL)**					
** Median (min-max)**	0.7 (0-7.4)	0.7 (0.4-1.1)	1.3 (0-3.9)	2.9 (0.2-7.4)	0.45 (0-0.9)
**Serum Immunofixation: Heavy chain, n (%)**					
** IgG**	12 (63.2)	3 (60)	3 (60)	4 (80)	2 (50)
** IgM**	2 (10.5)	2 (40)	0 (0)	0 (0)	0 (0)
** IgA**	3 (15.8)	0 (0)	1 (20)	1 (20)	1 (25)
** Negative**	2 (10.5)	0 (0)	1 (20)	0 (0)	1 (25)
**Serum Immunofixation: light chain, n (%)**					
** Kappa**	13 (68.4)	4 (80)	3 (60)	4 (80)	2 (50)
** Lambda**	6 (31.6)	1 (20)	2 (40)	1 (20)	2 (50)
**Free Lambda Light Chain (mg/L)**					
** Median (min-max)**	9.6 (1.8-832.4)	9.1 (4.1-22.3)	18.6 (2.9-260.2)	6.4 (1.8-161.0)	138.12 (5.8-832.4)
**Free Kappa Light Chain (mg/L)**					
** Median (min-max)**	18.3 (1.2-8159.0)	10.5 (8.6-181.7)	18.3 (14.1-469.2)	1201.4 (8.2-8159.0)	8.2 (1.2-3926.8)
**Serum Free Light Chain Ratio (i:U)**					
** Median (min-max)**	18.5 (0.9-1362.1)	1.1 (0.9-8.1)	18.5 (1.0-48.7)	186.8 (17.3-1362.1)	252.9 (2.6-723.8)
**IgA (mg/dL)**					
** Median (min-max)**	75 (2-1457)	118 (69-252)	154 (7-1457)	47 (14-59)	69 (2-516)
**IgG (mg/dL)**					
** Median (min-max)**	1089 (215-9033)	1113 (716-1364)	1159 (715-4940)	3388 (215-9033)	651 (269-1089)
**IgM (mg/dL)**					
** Median (min-max)**	27 (10-462)	160 (63-462)	30 (10-77)	18 (10-27)	12.5 (10-43)
**Type of Bone marrow, n (%)**					
** Unilateral (Left Side)**	7 (36.8)	1 (20)	1 (20)	2 (40)	3 (75)
** Unilateral (Right Side)**	8 (42.1)	4 (80)	4 (80)	0 (0)	0 (0)
** Bilateral**	4 (21.1)	0 (0)	0 (0)	3 (60)	1 (25)
**Plasma cell percentage in Right CORE**					
** Median (min-max)**	10 (0-100)	5 (4-7)	30 (5-40)	50 (30-100)	5 (0-10)
**Plasma cell percentage in the right aspirate**					
** Median (min-max)**	4 (0-76)	3 (1-4)	9 (4-10)	30 (15-76)	1 (0-3)
**Plasma cell percentage in LEFT CORE**					
** Median (min-max)**	7 (0-85)	5 (3-7)	30 (7-35)	57.5 (30-85)	0 (0-10)
**Plasma cell percentage in the LEFT aspirate**					
** Median (min-max)**	4 (0-53)	2 (1-5)	7 (4-10)	38.5 (24-53)	0 (0-2)
**FLOW Aberrant plasma cells from total analyzed (%)**					
** Median (min-max)**	92 (0-100)	40 (0-97)	84 (78.9-98)	100 (95.2-100)	76 (0-99)
**CD19, n (%)**					
** Positive (Dim)**	1 (5.3)	1 (20)	0 (0)	0 (0)	0 (0)
** Negative**	18 (94.7)	4 (80)	5 (100)	5 (100)	4 (100)
**CD27, n (%)**					
** Positive**	5 (26.3)	1 (20)	4 (80)	0 (0)	0 (0)
** Positive (Decreased)**	1 (5.3)	1 (20)	0 (0)	0 (0)	0 (0)
** Positive (partial)**	4 (21.1)	1 (20)	0 (0)	3 (60)	0 (0)
** Negative**	9 (47.4)	2 (40)	1 (20)	2 (40)	4 (100)
**CD38, n (%)**					
** Positive**	15 (78.9)	3 (60)	5 (100)	5 (100)	2 (50)
** Negative**	4 (21.1)	2 (40)	0 (0)	0 (0)	2 (50)
**CD45, n (%)**					
** Positive**	1 (5.3)	0 (0)	0 (0)	1 (20)	0 (0)
** Positive (Dim)**	4 (21.1)	2 (40)	0 (0)	2 (40)	0 (0)
** Positive (Partial)**	1 (5.3)	0 (0)	0 (0)	0 (0)	1 (25)
** Negative**	13 (68.4)	3 (60)	5 (100)	2 (40)	3 (75)
**CD56, n (%)**					
** Positive**	11 (57.9)	2 (40)	5 (100)	2 (40)	2 (50)
** Positive (Partial)**	2 (10.5)	1 (20)	0 (0)	1 (20)	0 (0)
** Negative**	6 (31.6)	2 (40)	0 (0)	2 (40)	2 (50)
**CD81, n (%)**					
** Positive (Partial)**	2 (10.5)	0 (0)	0 (0)	1 (20)	1 (25)
** Negative**	17 (89.5)	5 (100)	5 (100)	4 (80)	3 (75)
**CD117, n (%)**					
** Positive**	3 (15.8)	2 (40)	1 (20)	0 (0)	0 (0)
** Positive (Dim)**	2 (10.5)	0 (0)	0 (0)	1 (20)	1 (25)
** Positive (Partial)**	2 (10.5)	0 (0)	1 (20)	1 (20)	0 (0)
** Negative**	12 (63.2)	3 (60)	3 (60)	3 (60)	3 (75)
**CD138, n (%)**					
** Positive**	16 (84.2)	3 (60)	5 (100)	5 (100)	3 (75)
** Negative**	3 (15.8)	2 (40)	0 (0)	0 (0)	1 (25)
**cyKAPPA, n (%)**					
** Positive**	10 (52.6)	3 (60)	3 (60)	4 (80)	0 (0)
** Negative**	9 (47.4)	2 (40)	2 (40)	1 (20)	4 (100)
**cyLAMBDA, n (%)**					
** Positive**	4 (21.1)	0 (0)	2 (40)	1 (20)	1 (25)
** Negative**	15 (78.9)	5 (100)	3 (60)	4 (80)	3 (75)
**Karyotype, n (%)**					
** Normal**	11 (57.9)	3 (60)	5 (100)	1 (20)	2 (50)
** Abnormal**	3 (15.8)	0 (0)	0 (0)	2 (40)	1 (25)
** Hypodiploidy**	1 (5.3)	0 (0)	0 (0)	1 (20)	0 (0)
** Hyperdiploidy**	1 (5.3)	0 (0)	0 (0)	1 (20)	0 (0)
** Not done**	1 (5.3)	1 (20)	0 (0)	0 (0)	0 (0)
** Not ordered**	1 (5.3)	1 (20)	0 (0)	0 (0)	0 (0)
** Not Available**	1 (5.3)	0 (0)	0 (0)	0 (0)	1 (25)
**Alive (Yes/No), n (%)**					
** Yes**	10 (52.6)	3 (60)	4 (80)	3 (60)	0 (0)
** No**	7 (36.8)	0 (0)	1 (20)	2 (40)	4 (100)
** Unknown**	2 (10.5)	2 (40)	0 (0)	0 (0)	0 (0)
**Follow-up, days**					
** Median (min-max)**	1116 (213-2057)	1116 (599-1447)	1717 (399-1952)	1971 (213-2057)	583 (260-817)

### Cluster Analysis across all cells in the BMA

**Fig. 2:**
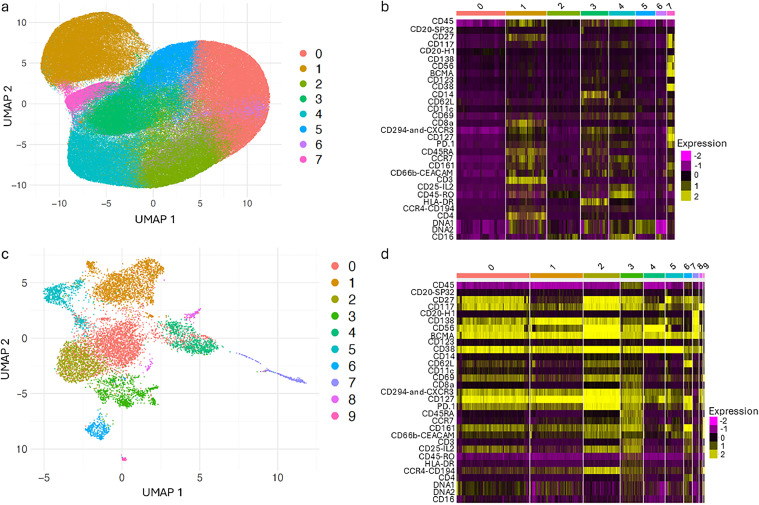
**Nested Clustering deconvolutes MM cell phenotypes in the BMA.** a) UMAP shows clustering of all analyzed cells using 5 principal components at a resolution of 0.2 creating 1 PC cluster and 7 immune cell clusters from the 8 unique primary clusters. b) Heatmap of the markers expressed across the 8 primary clusters. c) UMAP showing the reclustering of the PC cluster (cluster 7) from (a) using 25 principal components at a resolution of 0.2. d) Heatmap of markers expressed across all PCs from the nested clusters in (c).

A nested clustering approach of the multiplexed proteomic data deconvoluted distinct cellular subtypes/phenotypes within the BMA. The dataset comprises 289,289 cells from all 22 patients with an average of 13,149 (median = 12,685, range: 5271 – 28728) cells per patient. Initial clustering of all cells analyzed using only 5 principal components identified 8 primary clusters representing the major cell types (Fig. 2a). The number of cells per cluster varied across patients, reflecting an uneven distribution. Primary clusters 1 (*n* = 54,834; 19.0 %) and 7 (*n* = 10,420; 3.6 %) are separate from the other clusters, indicating distinct cellular phenotypes based on CD45-/CD3+ (T cells) and CD45-/CD38+/CD138+/CD56+/BCMA+ (PCs) expression, respectively (Fig. 2b). There is overlap between primary clusters 0 (*n* = 65,677; 22.7 %) and 2-6 suggesting that these clusters are not specific to a certain cellular phenotype. Cluster 4 (*n* = 35,369; 12.2 %) has consistently high CD45RO and CD16 expression, suggesting this may be an NK cell population expressing the memory-associated marker. Cluster 3 (*n* = 37,345; 12.9 %) has consistently high CD14 expression with moderate expression of CD45RA suggesting a more "naïve" or less differentiated subset of monocytes. Clusters 0, 2 (*n* = 44,738; 15.5 %), 5 (*n* = 26,523; 9.2 %), and 6 (*n* = 14,383; 5.0 %) are mixed populations of immune cells, within the boundary condition of the selected markers. The detection of primary clusters consisting of the TiME and PCs is consistent with the goals of the study.

### Plasma Cell Landscape in MM

Next, sub-clustering of primary cluster 7 representing the PC population identified 10 unique subtypes across the disease states, suggesting that PCs consist of several different and phenotypically well-defined cell subpopulations (Fig. 2, Fig. 2). Interestingly, CD45 and CD138 expressions varied across clusters, revealing three distinct PC categories based on their expression levels. PC clusters 3 (*n* = 977) and 6 (*n* = 344) exhibited moderate CD45 and low/moderate CD138 expression (CD45 pos/CD138 low), whereas PC clusters 4 (*n* = 907), 5 (*n* = 774), 8 (*n* = 144), and 9 (*n* = 52) displayed no /low CD45 and low/moderate CD138 expression (CD45 low/CD138 low). Meanwhile, PC clusters 0 (*n* = 3129), 1 (*n* = 2269), 2 (*n* = 1562), and 7 (*n* = 262) exhibited no CD45 and high CD138 expression (CD45 neg/CD138 high) (Fig. 2d). Given the variability across patients, it is possible that these differences reflect patient-specific influences, particularly depending on the representation of each patient within a given cluster.

**Fig. 3:**
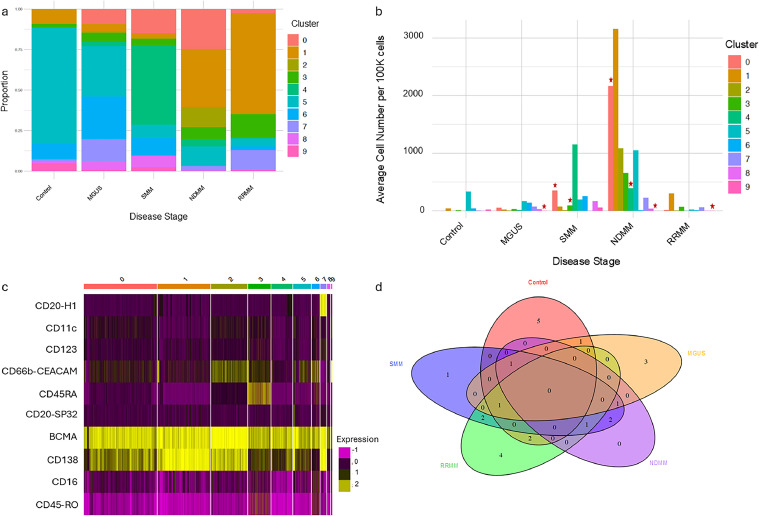
**PC subtype distribution in different disease states and controls.** a) Proportions of PC clusters in each disease state. b) Average cell numbers in each cluster per 100 K cells in each disease state. (*) clusters significantly different compared to control. c) Heatmap for the top 10 variable biomarkers across PC clusters. d) Venn-diagram representing differentially expressed markers in each disease state compared to all other groups.

Next, the PC clusters were analyzed by disease state to show the unique PC profiles across disease progression (Fig. 3a). There was an observable difference in the abundance of each PC cluster across the different disease states (Fig. 3b). Interestingly, we found that the PC clusters 6 (CD45 pos/CD138 low), 4 and 5 (CD45 low/ CD138 low) represent the top abundant clusters in the control and the precursor states. In contrast, the PC clusters 0, 1, and 2 (CD45 neg/CD138 high) dominated NDMM (Fig. 3a). This pattern suggests a progressive evolution of PCs along a spectrum of states, with a progressive shift from CD45 pos/CD138 low to CD45 neg/CD138 high through a transition state (CD45 low/ CD138 low).

PC clusters identified by CD138 and BCMA had cluster-specific differences in additional markers (Fig. 3c, Table 3). Interestingly, control samples predominantly exhibited PC cluster 5 which had high CD66b, CD27, and CD38 expression with variable (low to high) CD138 and BCMA expression. The proportion of this cluster was observed to diminish with disease progression. Similarly, PC cluster 9 (CD66b high) decreased significantly from the controls with minimal presence in precursor disease to overt disease states. MGUS and SMM displayed emerging clusters, such as PC cluster 0 which was defined by high BCMA, CD25, CD117, and CD27 expression with variable (low to high) CD138 expression and variable (little to none) CD11c and CD66b expression profiles. The NDMM state presented with nearly all PC clusters with the most balanced distribution of subtypes. Both NDMM and RRMM were dominated by PC cluster 1 which was elevated compared to control and precursor states. PC cluster 1 had high BCMA, CD127, CD38, and CD138 expression with variable (no to low) CD66b. This shows that the PC subtype distribution changes with disease states.

**Table 3:** **Highest expression of markers in each PC Cluster showing changes across the disease states.** Only markers identified to have a significant increase in expression in each cluster compared to all other cells are provided. Significance defined as p-value < 0.05.

PC Cluster 0	PC Cluster 1	PC Cluster 2	PC Cluster 3	PC Cluster 4	PC Cluster 5	PC Cluster 6	PC Cluster 7	PC Cluster 8	PC Cluster 9
CD25	CD138	PD.1	CD45RA	CD56	CD38	CD4	CD20-H1	CD117	CD66b
CD117	CD127	CD56	CD3	CD38	CD27	CD161	CD138	HLA-DR	CCR7
CD27	BCMA	CD27	HLA-DR		CD66b	CD62L	CD56		CCR4
	CD38	CD25-IL2	CD45			CD45-RO	CD123		CD11c
			CD8a			CD45			CD69

No clusters uniquely existed in any state; however, differential expression analysis (27,000 to 77,000 cells/disease state) identified state-unique markers when comparing each state to all others (Fig. 3d). Specifically, CD294 and CXCR3 for MGUS, PD1 for SMM, and CD16, CD66b, CD27, and CD45RO for RRMM, while no unique markers were detected for NDMM. The absence of state-specific clusters suggests that while these markers may distinguish disease states at the expression level, they were not the primary drivers of clustering. This indicates that PC subtypes across states share broader phenotypic similarities, which may have influenced the clustering outcome.

### Immune landscape of multiple myeloma

**Fig. 4:**
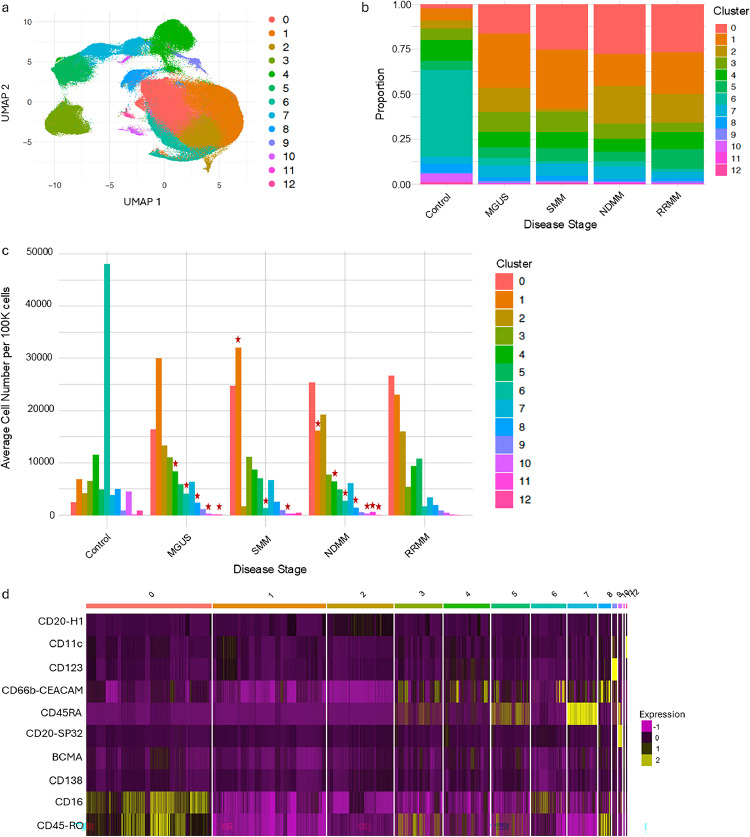
**Resolving TiME heterogeneity across MM disease states.** a) UMAP showing clustering of non-PCs using 25 PCAs at a resolution of 0.2. b) Proportions of stroma clusters in each disease state. c) Average cell numbers in each cluster per 100k cells in each disease state. (*) clusters significantly different compared to control. d) Heatmap shows the expression of all biomarkers across TiME clusters.

To characterize the tumor immune microenvironment (TiME) of MM, clustering analyses were performed on primary clusters 0-6 (Fig. 2a) yielding 13 distinct TiME clusters (Fig 4a). Clusters 3, 10, and 12 are distinct from the other clusters possibly representing unique cellular phenotypes. There is a small portion of overlap between Cluster 4 and Clusters 7, 8, and 9, while Cluster 5 also overlaps with Cluster 7 indicating potential phenotypic similarities between these cells. Clusters 0, 1, 2, 6, and 11 all overlap, encompassing a large region within the UMAP indicating many commonalities shared between these cells.

Comparative TiME cluster analyses revealed that there was not a single cluster exclusively present in any disease state. However, the immune landscape of all four MM states differs substantially from the control group, with relatively minor differences observed between individual disease states (Fig. 4, Fig. 4). Specific clusters (Cluster IDs: 4, 6, 8, 10, and 12) were notably decreased across all MM states compared to controls, whereas Cluster 11 was significantly increased exclusively in NDMM. Cluster 6 was the most dominant cell phenotype in the control group with the most significant reduction across precursor states and NDMM (Fig 4c).Interestingly, marker analysis of individual clusters indicated that Clusters 4 and 6 appeared to represent monocyte/macrophages, while Clusters 8, 10, and 12 were likely composed of T cells, B cells, and dendritic monocytes, respectively (Table 4). This indicates a change in the distribution of TiME subtypes from non-MM to MM disease states.

**Table 4:** **Highest expressed biomarkers in non-PC TiME clusters that show significant changes across MM states compared to control samples.** Only markers identified to have a significant increase in expression in each cluster compared to all other cells are provided. Significance defined as p-value < 0.05.

Cluster 0	Cluster 1	Cluster 2	Cluster 3	Cluster 4	Cluster 5	Cluster 6	Cluster 7	Cluster 8	Cluster 9	Cluster 10	Custer 11	Cluster 12
CD45RO	DNA1	CD20	CD4	CD14	CD8a	CD62L	CD161	CCR7	CD123	CD20	BCMA	CD11c
CD16	DNA2	CD138	CD3	HLA-DR	CD3	CD66b	CCR7	CD294 and CXCR3	CD11c	CD45RA	CD138	CD123
CD25		CD56	CD27	CD127	CD45RA	CD16	HLA-DR	CD66b	CD294 and CXCR3	HLA-DR	CD127	CD20
CD45			CD194		CCR7	CD20		CD45RO	HLA-DR	CD62L		
CD69			CD127		CD127			CD25	CD38	CD16		

Interestingly, Cluster 1 was elevated across MM states compared to controls with MGUS and SMM precursor states having a higher frequency and incidence than NDMM and RRMM. Clusters 1, 2, and 11 had little to no marker expression across cells suggesting they represent various populations of cells undefined by the proteomic panel used in this study. Interestingly, Clusters 2 and 11 may include some PCs given the low expression of CD20 and CD138, consistent with its significant increase in NDMM. These findings underscore the profound alterations in the TiME in different MM states and highlight the potential roles of specific immune subsets, including monocytes/macrophages and T and B cell populations, in disease pathogenesis.

### Clinical correlation and survival analysis

The potential clinical significance of the BMA analytes detected across disease states was explored through correlations between the PC and TiME clusters and clinical data elements using the Wilcoxon rank sum test for categorical variables and Spearman’s rank correlation for continuous and ordinal variables. There were no significant correlations based on individual disease states, which may be due to the limited sample size per cohort. Further analysis by precursor or overt disease states, or taking all samples together showed significant correlations between cell types and clinical metrics. All significant correlations are presented in **Supplemental Table 2**.

PC cluster 0 (CD45 neg/CD138 high) was the most common PC phenotype to correlate with either the clinical or TiME Clusters in precursor states, accounting for >50 % of the significant correlations. There were no correlations between PC clusters observed in either precursor or overt disease states. Interestingly, in the precursor states there was a correlation between TiME clusters as well as between PC and TiME clusters, while this was not observed in overt disease states. Together this highlights the role of TiME in early disease formation. Minimal significant correlations were observed in overt disease states. CD117 expression was the most common variable correlating with PC and TiME clusters, interestingly PC cluster 3 and TiME cluster 0 were positively correlated while PC cluster 0 and TiME cluster 2 were negatively correlated suggesting a complex interplay between cellular phenotypes.

Analysis across all samples showed that PC cluster 0 was the most common PC phenotype to correlate across the disease states with clinical flow cytometry plasma cell percentages (aberrant and total). PC cluster 0 also correlated with PC clusters 4 and 8, suggesting a potential relationship between these phenotypes as disease progresses. Interestingly, PC clusters 0,1, 2 and TiME cluster 9 all correlated with aberrant plasma cell percentage as determined by clinical flow cytometry. TiME cluster 9 was the most common TiME phenotype to correlate across the disease states, having a negative correlation with PC percentage (aberrant and total).

We further conducted a survival analysis to reveal significant associations between specific clusters and overall survival (OS; **Supp Fig 1**). OS was determined by alive status, unfortunately 1 SMM, 2 NDMM, and 4 RRMM patients passed away since sample collection. Given the single patient in the SMM cohort with an event, this analysis was not powered for precursor disease state outcomes. RRMM did not have any significant associations between OS time and liquid biopsy cell clusters. OS was associated with PC Clusters 1, 4, and 8, as well as TiME Clusters 0, 10, 3, 4, 8, and 9 in NDMM. Interestingly, when combining analysis across overt disease states, PC Cluster 5 was also found to be significantly associated with OS. Taken together this data indicates a complex interplay between PCs and the TiME that influences survival outcomes.

## Discussion

This study provides a comprehensive analysis of cellular proteomics of PC disorders at varying states, highlighting the dynamic interplay between cancer cell subtypes, state-specific cluster abundance, and the evolving immune landscape of the bone marrow microenvironment. By leveraging multiplexed targeted proteomics via high-resolution IMC, we characterized the complexity of PCs and the TiME across distinct MM states, offering critical insights into the biological and clinical implications of MM progression. There are 2 key findings: 1) changes in the PC and TiME landscape between MM states, and 2) state-specific PC subtypes with unique biomarker expression profiles.

Our findings demonstrate that PC subtype distribution in MM changes with disease states, suggesting the evolution of PC subtypes with disease progression. This expands the biological understanding of PC subtypes described in our previous study [16]. Our results are consistent with prior work using sophisticated technologies such as single-cell global transcriptomic, genomic and proteomics analyses, which showed that the abundance of abnormal PCs and the emergence of specific subtypes increase as the disease advances [24,[26], [27], [28]]. Notably, our ability to replicate these findings using a simpler IMC-based proteomic assay on liquid biopsy highlights its clinical utility and applicability for individualized patient assessment. Moreover, the compatibility of IMC with slide based liquid biopsy enables not only the enumeration of abnormal PCs, but also the identification of single-cell genetic lineages and proteomic phenotypes.

Using a nested clustering approach on multiplexed proteomic data, we identified 10 unique PC subtypes across MGUS, SMM, NDMM, and RRMM, with varying marker expression profiles. Notably, no single cluster was exclusive to any disease stage, yet certain markers were distinctly associated with specific states. Prior genomic analyses have similarly highlighted the presence of distinct PC subtypes in MM compared to healthy donors with identified molecular signatures and functional variations among PC subtypes [19,20]. Importantly, state-specific markers were identified that may aid in diagnosis and monitoring. For instance, CD294 and CXCR3 were associated with MGUS and PD1 with SMM. These findings suggest that PC diversity may contribute to MM pathogenesis, underscoring the need for further investigations into how these subtypes influence disease progression and potentially therapeutic response.

One of the key observations was the progressive loss of CD45 pos/CD138 low and CD45 low/ CD138 low PC clusters 4, 5, 6, and 9, which were prevalent in control and precursor samples but significantly diminished with disease advancement. This suggests that early PC subtypes may either evolve into unique phenotypes or be selectively eliminated as the disease progresses. The emergence of CD45 neg/CD138 high PCs clusters 0, 1 and 2 in disease progression supports the notion that MM is shaped by the dynamic evolution of PCs. Notably, NDMM presented with nearly all PC clusters reflecting the intricacy of the disease at this stage. The complex proteogenomic landscape of NDMM has been described previously [21] and the loss of CD45 expression has been associated with worse survival [22]. In contrast, RRMM was less diverse, dominated by cluster 1, potentially indicating the selective pressure exerted by therapy on tumor cells.

Our data suggest the existence of developmental relationships between different PC subgroups. Beyond the shift from CD45 high/CD138 low to CD45 low/CD138 high pattern, several PCs clusters expressed immune cell markers in addition to canonical PC markers. This pattern may further support an evolutionary trajectory of PCs—from normal to early and then late malignant stages. For instance, clusters 3 and 6 expressed T cell markers such as CD3, CD4, and CD62L, alongside aberrant PC markers including CD138, CD38, and CD27 (a B cell marker). These findings raise the possibility that these cells represent an early PC lineage, a T cell-like phenotype, or even an innate-like cell state. Another notable example is cluster 2, which displayed high PD-1 expression. If these cells also express PD-L1, it would suggest the formation of an autocrine signaling loop that promotes tumor cell survival and proliferation—a potential mechanism for immune evasion and therapeutic resistance in advanced disease. Future studies incorporating PD-L1 into the IMC panel will be critical to explore the functional relevance of this cluster.

The co-expression of PC and immune cell markers could also reflect physical interactions between PCs and immune cells as part of the host immune response. Particularly intriguing is cluster 3, which co-expresses both PC and T cell markers. This warrants further investigation into tumor–immune cell interactions that may shape MM progression. Finally, it is important to consider the possibility that these mixed phenotypes arise from clustering limitations due to the relatively low number of PCs in each sample, especially in precursor states where PCs comprise <10 % of the total cell population. Future studies incorporating larger cell numbers will be essential to improve clustering resolution and confidently capture rare PC populations.

The study also sheds light on the dynamic changes in the TiME landscape of the bone marrow between MM disease states. Clustering analyses of non-PC populations revealed 13 distinct TiME clusters, with significant alterations observed across MM stages compared to the control. Definitive phenotyping of these clusters was somewhat limited by the relatively small number of markers included in the IMC panel. To address this, we identified the top upregulated markers in each cluster (Table 4) relative to all others, aiding in the classification of most clusters. For instance, cluster 2 (CD20, CD138, and CD56) likely represents early-stage abnormal PCs; cluster 3 (CD4, CD3, CD27, CD194, CD127) resembles effector memory or Th2-like CD4⁺ T cells; cluster 4 (CD14 and HLA-DR) most likely corresponds to activated monocytes; and cluster 5 (CD8, CD3, CD45RA) is suggestive of naïve CD8⁺ T cells. In contrast, some clusters displayed mixed phenotypes. For example, cluster 6 exhibited upregulation of both T cell markers (CD62L, CD66b, CD16) and B cell markers (CD20), indicating a transitional population. Future studies incorporating a broader set of markers and analyzing larger cell numbers will be essential to enable more definitive phenotyping.

Our findings showed that specific immune clusters (e.g., Clusters 4, 6, 8, 10, and 12) were substantially reduced across all MM stages, reflecting the profound immunosuppressive environment characteristic of MM. Notably, cluster 6, which was dominant in the control, diminished significantly with disease progression. These clusters likely represent key immune subsets, including monocytes/macrophages, T cells, B cells, and dendritic cells. Despite the partial uncertainty of the identity of some of these clusters, our findings highlight the immune dysregulation that occurs during MM progression and underscore the critical roles of specific immune phenotypes and adaptive immune populations in shaping the TiME, which has been observed in other studies [[23], [24], [25]]. These dynamic shifts not only provide insights into disease pathogenesis but also present opportunities to modulate the immune response. A key question remains: does the TiME drive PC changes, or do PCs shape the TiME? Understanding this interplay will be critical for developing targeted therapeutic strategies.

While our study provides valuable insights into the distribution of bone marrow PCs and the immune landscape in MM, several limitations must be acknowledged. Although IMC enables spatial proteomics analyses, this aspect was not captured in our study due to the use of BMAs, which do not preserve spatial architecture. Our study utilized BMAs because they are more routinely collected and available from patients across all states of MM and control individuals, making them more broadly representative. Our analysis focused on profiling the phenotypic diversity and abundance of PCs and TiME across disease states, which remains highly informative. Still, future IMC analyses of bone marrow trephine biopsies can overcome the spatial limitation of BMAs, enabling the in-situ examination of interactions between malignant PCs and the surrounding microenvironment. Such spatial insights could significantly enhance our understanding of TiME role in shaping MM heterogeneity and evolution. Moreover, our study focused on proteomic profiling of single-cell populations; integrating complementary genomic and transcriptomic data could further refine our understanding of the MM landscape. Additionally, the small cohort with moderate follow-up time restricts our ability to correlate findings with long-term clinical outcomes, such as survival. Future longitudinal studies will be essential for determining the prognostic relevance of specific PC and TiME subsets, with time-resolved MGUS analyses providing insights into cellular population dynamics and disease progression. Despite these limitations, our findings provide a comprehensive characterization of PC subtypes and TiME alterations in MM, reinforcing the need for a multi-faceted approach to disease monitoring and treatment. Future studies leveraging multi-omic analyses and functional validation of identified PC and immune subsets will be instrumental in translating these findings into clinical applications aimed at improving MM patient outcomes.

Overall, this study underscores the profound change of PCs and TiME cellular distributions in the bone marrow in different MM disease states. The identification of stage-specific cell clusters and markers, coupled with a deeper understanding of TiME alterations, offers valuable insights into MM biology. These findings have significant implications for early diagnosis, disease monitoring, and the development of targeted therapeutic strategies. Future longitudinal studies are warranted to further elucidate these dynamic changes and their potential as predictive biomarkers or therapeutic targets.

## Data availability statement

All data discussed in this manuscript are included in the main manuscript text or supplementary materials. The proteomics data are available through the BloodPAC Data Commons, Accession ID “BPDC000148” (https://data.bloodpac.org/discovery/BPDC000148/).

## Funding

This work was funded in whole or in part by the Dr. Miriam and Sheldon G. Adelson Medical Research Foundation (P.K., R.O.), the NIH/NCI U01CA258013 (P.K., J.M., S.N.S.), Epic Sciences (P.K.), and USC Norris Comprehensive Cancer Center (CORE) Support P30CA014089 (P.K., J.M.). This work also received institutional support from the USC Michelson Center Convergent Science Institute in Cancer, Vassiliadis Research Fund, and Hart Family Research Fund. The content is solely the responsibility of the authors and does not necessarily represent the official views of the National Institutes of Health.

## CRediT authorship contribution statement

**Mohamed Kamal:** Conceptualization, Data curation, Formal analysis, Validation, Visualization, Writing – original draft, Writing – review & editing. **Stephanie N. Shishido:** Conceptualization, Formal analysis, Investigation, Project administration, Validation, Visualization, Writing – original draft, Writing – review & editing. **Jeremy Mason:** Conceptualization, Data curation, Formal analysis, Investigation, Validation, Visualization, Writing – original draft, Writing – review & editing. **Krina Patel:** Resources, Writing – review & editing. **Elisabet E. Manasanch:** Funding acquisition, Resources, Writing – review & editing. **Robert Z. Orlowski:** Funding acquisition, Resources, Writing – review & editing. **Peter Kuhn:** Conceptualization, Funding acquisition, Project administration, Writing – review & editing.

## Declaration of competing interest

The authors declare that they have no known competing financial interests or personal relationships that could have appeared to influence the work reported in this paper.

## Appendix Supplementary materials

Image, application 1
