# Corrigendum to “Single-cell proteomic analysis reveals Multiple Myeloma heterogeneity and the dynamics of the tumor immune microenvironment in precursor and advanced states” [Neoplasia 66 (2025) 1101189]

**DOI:** 10.1016/j.neo.2025.101226

**Published:** 2025-09-06

**Authors:** Mohamed Kamal, Stephanie N. Shishido, Jeremy Mason, Krina Patel, Elisabet E. Manasanch, Robert Z. Orlowski, Peter Kuhn

**Affiliations:** a Convergent Science Institute for Cancer, Michelson Center, University of Southern California, Los Ange-les CA 90089, USA; b Department of Lymphoma and Myeloma, USA; c Department of Experimental Therapeutics, Division of Cancer Medicine, University of Texas MD Anderson Cancer Center, Houston, TX, 77030, USA; d Catherine & Joseph Aresty Department of Urology, Institute of Urology, Keck School of Medicine, University of Southern California, Los Angeles, CA 90033, USA; e Norris Comprehensive Cancer Center, Keck School of Medicine, University of Southern California, Los Angeles CA 90033, USA; f Department of Biomedical Engineering, Viterbi School of Engineering, University of Southern California, Los Angeles, CA 90089, USA; g Department of Aerospace and Mechanical Engineering, Viterbi School of Engineering, University of Southern California, Los Angeles, CA 90089, USA; h Department of Biological Sciences, Dornsife College of Letters, Arts, and, Sciences, University of Southern California, Los Angeles, CA 90089, USA

The authors regret discovering that one of the markers **(CD20)** had been inadvertently omitted from the staining of 15 samples. Although CD20 was expressed in only a small minority of cells, we nonetheless reanalysed the data after removing CD20 from all samples. We affirm that none of these corrections alter the study’s scientific conclusions. Importantly, the main findings of the study remain unchanged.

The details of the corrected analysis after excluding CD20 from the dataset are as follows:

1. The initial primary clustering structure remained unchanged (Figure 2a and b).
2. After re-clustering the PC enriched cluster (primary cluster 7) and the rest of the clusters (TiME) independently, the number of identified clusters changed slightly:
  ○ PC clusters from primary cluster 7 decreased from 10 to 9
  ○ TiME clusters decreased from 13 to 12

This affected the lists of top upregulated markers in each cluster but did not alter the overall cluster marker profiles reported in Figures 2D and 4D of the published paper or the key findings of the study.

Cell distribution per cluster:

Minor shifts in cluster labels and cell counts per cluster for both PC and TiME were observed due to the updated analysis.

3. State-specific PC markers update:

The previously reported state-specific markers identified by differential expression changed slightly after recalculation from those reported in Figure 3D of the paper (See table below).

**Table:** 

**Disease State**	**Original Analysis**	**adj p-value**	**CD20-excluded Analysis**	**adj p-value**
**MGUS**	CD294–CXCR3	3.80 × 10⁻⁷⁶	CD294–CXCR3	9.65 × 10⁻⁹⁶
			CD45	7.47 × 10⁻¹³
			CD4	3.89 × 10⁻²

**SMM**	PD1	1.53 × 10⁻¹²⁸	PD1	9.90 × 10⁻¹²⁶
			CD45RA	2.15 × 10⁻⁹⁵

**RRMM**	CD16	2.44 × 10⁻⁷⁰	CD16	1.16 × 10⁻⁸⁶
	CD66b	1.29 × 10⁻³⁸		
	CD27	9.51 × 10⁻³¹		
	CD45RO	3.08 × 10⁻²⁹	CD45RO	7.74 × 10⁻²⁹

4. Image Preprocessing methods section:

We also note a clarification to the Image Preprocessing methods section:

*“CD28 and CD223 did not pass quality control checks and were excluded from downstream analyses*

The authors would like to apologise for any inconvenience caused.

