# Supplementary material for: Single-cell proteomic analysis reveals Multiple Myeloma heterogeneity and the dynamics of the tumor immune microenvironment in precursor and advanced states
Source: Neoplasia. 2025 Jun 6;66:101189. doi: 10.1016/j.neo.2025.101189 (PMC12173141; doi:10.1016/j.neo.2025.101189)

**Supplemental Information**

**Supplemental Table 1: Cell count per PC and TiME cluster.**

| Cluster | Total Cells |
| --- | --- |
| Primary 0 | 65677 |
| Primary 1 | 54834 |
| Primary 2 | 44738 |
| Primary 3 | 37345 |
| Primary 4 | 35369 |
| Primary 5 | 26523 |
| Primary 6 | 14383 |
| Primary 7 | 10420 |
| PC 0 | 3129 |
| PC 1 | 2269 |
| PC 2 | 1562 |
| PC 3 | 977 |
| PC 4 | 907 |
| PC 5 | 774 |
| PC 6 | 344 |
| PC 7 | 262 |
| PC 8 | 144 |
| PC 9 | 52 |
| TiME 0 | 66550 |
| TiME 1 | 60038 |
| TiME 2 | 35147 |
| TiME 3 | 25251 |
| TiME 4 | 24555 |
| TiME 5 | 20427 |
| TiME 6 | 18623 |
| TiME 7 | 15704 |
| TiME 8 | 6663 |
| TiME 9 | 2494 |
| TiME 10 | 1991 |
| TiME 11 | 771 |
| TiME 12 | 655 |

**Supplemental Table 2: Significant correlations between cell clusters and clinical data.** Wilcoxon = Wilcoxon Rank Sum. Significance defined as p-value < 0.05.

| Level | Cluster | Clinical Variable or Cluster | Correlation Type | Correlation Value | p-Value |
| --- | --- | --- | --- | --- | --- |
| All | PC 0 | cyKAPPA | Wilcoxon | -3.35 | 0.0008 |
| All | PC 0 | Plasma cell percentage in Right CORE | Spearman | 0.69 | 0.0010 |
| All | PC 0 | Plasma cell percentage in the Right aspirate | Spearman | 0.69 | 0.0010 |
| All | PC 0 | PC 8 | Spearman | 0.66 | 0.0022 |
| All | PC 0 | Plasma cell percentage in Left CORE | Spearman | 0.71 | 0.0030 |
| All | PC 0 | PC 4 | Spearman | 0.63 | 0.0037 |
| All | PC 0 | Free Kappa Light Chain (mg/L) | Spearman | 0.63 | 0.0038 |
| All | PC 0 | CD138 | Wilcoxon | -2.46 | 0.0139 |
| All | PC 0 | TiME 11 | Spearman | 0.55 | 0.0139 |
| All | PC 0 | CD38 | Wilcoxon | -2.40 | 0.0164 |
| All | PC 0 | FLOW Aberrant plasma cells from total analyzed (%) | Spearman | 0.53 | 0.0205 |
| All | PC 0 | CD27 | Wilcoxon | -2.29 | 0.0222 |
| All | PC 0 | CD56 | Wilcoxon | -2.19 | 0.0283 |
| All | PC 0 | Plasma cell percentage in the Left aspirate | Spearman | 0.53 | 0.0425 |
| All | PC 1 | TiME 8 | Spearman | -0.61 | 0.0057 |
| All | PC 1 | CD138 | Wilcoxon | -2.46 | 0.0139 |
| All | PC 1 | TiME 9 | Spearman | -0.54 | 0.0163 |
| All | PC 1 | TiME 12 | Spearman | -0.52 | 0.0217 |
| All | PC 1 | FLOW Aberrant plasma cells from total analyzed (%) | Spearman | 0.49 | 0.0319 |
| All | PC 2 | PC 0 | Spearman | 0.67 | 0.0016 |
| All | PC 2 | PC 4 | Spearman | 0.62 | 0.0044 |
| All | PC 2 | Serum Free Light Chain Ratio (i:U) | Spearman | 0.59 | 0.0074 |
| All | PC 2 | Free Kappa Light Chain (mg/L) | Spearman | 0.59 | 0.0076 |
| All | PC 2 | Plasma cell percentage in Right CORE | Spearman | 0.57 | 0.0110 |
| All | PC 2 | FLOW Aberrant plasma cells from total analyzed (%) | Spearman | 0.51 | 0.0248 |
| All | PC 2 | PC 3 | Spearman | 0.51 | 0.0264 |
| All | PC 2 | TiME 9 | Spearman | -0.50 | 0.0293 |
| All | PC 2 | CD56 | Wilcoxon | -2.10 | 0.0353 |
| All | PC 2 | Plasma cell percentage in Left CORE | Spearman | 0.53 | 0.0445 |
| All | PC 2 | PC 8 | Spearman | 0.46 | 0.0472 |
| All | PC 3 | TiME 5 | Spearman | 0.55 | 0.0146 |
| All | PC 3 | Plasma cell percentage in Left CORE | Spearman | 0.61 | 0.0155 |
| All | PC 3 | PC 4 | Spearman | 0.50 | 0.0275 |
| All | PC 3 | TiME 0 | Spearman | 0.48 | 0.0363 |
| All | PC 3 | Serum Free Light Chain Ratio (i:U) | Spearman | 0.47 | 0.0425 |
| All | PC 4 | Plasma cell percentage in Left CORE | Spearman | 0.77 | 0.0009 |
| All | PC 4 | PC 8 | Spearman | 0.65 | 0.0026 |
| All | PC 4 | Plasma cell percentage in Right CORE | Spearman | 0.64 | 0.0034 |
| All | PC 4 | PC 0 | Spearman | 0.63 | 0.0037 |
| All | PC 4 | PC 3 | Spearman | 0.50 | 0.0275 |
| All | PC 4 | Plasma cell percentage in the Right aspirate | Spearman | 0.48 | 0.0368 |
| All | PC 4 | TiME 11 | Spearman | 0.46 | 0.0479 |
| All | PC 5 | TiME 1 | Spearman | 0.47 | 0.0440 |
| All | PC 6 | TiME 1 | Spearman | -0.50 | 0.0276 |
| All | PC 6 | TiME 6 | Spearman | -0.47 | 0.0418 |
| All | PC 6 | IgA (mg/dL) | Spearman | 0.47 | 0.0436 |
| All | PC 7 | TiME 2 | Spearman | 0.75 | 0.0002 |
| All | PC 7 | TiME 9 | Spearman | -0.58 | 0.0085 |
| All | PC 7 | TiME 5 | Spearman | -0.54 | 0.0175 |
| All | PC 7 | TiME 11 | Spearman | 0.53 | 0.0203 |
| All | PC 8 | TiME 11 | Spearman | 0.66 | 0.0022 |
| All | PC 8 | PC 0 | Spearman | 0.66 | 0.0022 |
| All | PC 8 | PC 4 | Spearman | 0.65 | 0.0026 |
| All | PC 8 | cyKAPPA | Wilcoxon | -2.45 | 0.0143 |
| All | PC 8 | Plasma cell percentage in the Right aspirate | Spearman | 0.54 | 0.0164 |
| All | PC 8 | CD38 | Wilcoxon | -2.40 | 0.0164 |
| All | PC 8 | Plasma cell percentage in Right CORE | Spearman | 0.51 | 0.0258 |
| All | PC 8 | Plasma cell percentage in Left CORE | Spearman | 0.56 | 0.0297 |
| All | PC 8 | TiME 4 | Spearman | -0.49 | 0.0327 |
| All | PC 8 | CD117 | Wilcoxon | -2.11 | 0.0346 |
| All | PC 8 | CD27 | Wilcoxon | -2.04 | 0.0412 |
| All | PC 8 | CD138 | Wilcoxon | -2.01 | 0.0442 |
| All | PC 9 | TiME 8 | Spearman | 0.58 | 0.0095 |
| All | PC 9 | PC 1 | Spearman | -0.56 | 0.0123 |
| All | PC 9 | TiME 3 | Spearman | 0.54 | 0.0173 |
| All | PC 9 | TiME 9 | Spearman | 0.53 | 0.0186 |
| All | PC 9 | PC 8 | Spearman | -0.46 | 0.0453 |
| All | TiME 0 | TiME 1 | Spearman | -0.62 | 0.0043 |
| All | TiME 0 | CD117 | Wilcoxon | 2.28 | 0.0225 |
| All | TiME 0 | Serum Free Light Chain Ratio (i:U) | Spearman | 0.51 | 0.0273 |
| All | TiME 0 | TiME 6 | Spearman | -0.49 | 0.0334 |
| All | TiME 0 | PC 3 | Spearman | 0.48 | 0.0363 |
| All | TiME 0 | CD38 | Wilcoxon | 2.00 | 0.0455 |
| All | TiME 1 | TiME 6 | Spearman | 0.64 | 0.0030 |
| All | TiME 1 | TiME 0 | Spearman | -0.62 | 0.0043 |
| All | TiME 1 | PC 6 | Spearman | -0.50 | 0.0276 |
| All | TiME 1 | PC 5 | Spearman | 0.47 | 0.0440 |
| All | TiME 10 | TiME 8 | Spearman | 0.67 | 0.0018 |
| All | TiME 11 | Free Lambda Light Chain (mg/L) | Spearman | -0.69 | 0.0010 |
| All | TiME 11 | PC 8 | Spearman | 0.66 | 0.0022 |
| All | TiME 11 | Plasma cell percentage in the Right aspirate | Spearman | 0.62 | 0.0043 |
| All | TiME 11 | cyKAPPA | Wilcoxon | -2.53 | 0.0114 |
| All | TiME 11 | PC 0 | Spearman | 0.55 | 0.0139 |
| All | TiME 11 | CD38 | Wilcoxon | -2.40 | 0.0164 |
| All | TiME 11 | Plasma cell percentage in Right CORE | Spearman | 0.53 | 0.0209 |
| All | TiME 11 | TiME 9 | Spearman | -0.51 | 0.0249 |
| All | TiME 11 | TiME 4 | Spearman | -0.51 | 0.0267 |
| All | TiME 11 | Type of Bone Marrow | Wilcoxon | -2.06 | 0.0390 |
| All | TiME 11 | PC 4 | Spearman | 0.46 | 0.0479 |
| All | TiME 12 | Type of Bone Marrow | Wilcoxon | 2.81 | 0.0050 |
| All | TiME 12 | PC 1 | Spearman | -0.52 | 0.0217 |
| All | TiME 12 | TiME 9 | Spearman | 0.46 | 0.0461 |
| All | TiME 2 | TiME 9 | Spearman | -0.64 | 0.0030 |
| All | TiME 3 | Type of Bone Marrow | Wilcoxon | 2.64 | 0.0082 |
| All | TiME 3 | TiME 9 | Spearman | 0.54 | 0.0182 |
| All | TiME 3 | IgM (mg/dL) | Spearman | 0.52 | 0.0233 |
| All | TiME 4 | Free Kappa Light Chain (mg/L) | Spearman | -0.52 | 0.0211 |
| All | TiME 4 | cyKAPPA | Wilcoxon | 2.29 | 0.0222 |
| All | TiME 4 | TiME 11 | Spearman | -0.51 | 0.0267 |
| All | TiME 4 | PC 8 | Spearman | -0.49 | 0.0327 |
| All | TiME 4 | Serum Immunofixation: light chain | Wilcoxon | -2.02 | 0.0437 |
| All | TiME 5 | PC 3 | Spearman | 0.55 | 0.0146 |
| All | TiME 6 | CD45 | Wilcoxon | -2.98 | 0.0029 |
| All | TiME 6 | TiME 1 | Spearman | 0.64 | 0.0030 |
| All | TiME 6 | TiME 0 | Spearman | -0.49 | 0.0334 |
| All | TiME 6 | PC 6 | Spearman | -0.47 | 0.0418 |
| All | TiME 7 | TiME 9 | Spearman | 0.55 | 0.0149 |
| All | TiME 7 | Mspike Value (SPEP) (gm/dL) | Spearman | 0.50 | 0.0283 |
| All | TiME 7 | Serum Immunofixation | Wilcoxon | 0.50 | 0.0283 |
| All | TiME 7 | Follow-up days | Spearman | 0.48 | 0.0397 |
| All | TiME 7 | IgG (mg/dL) | Spearman | 0.47 | 0.0413 |
| All | TiME 8 | TiME 10 | Spearman | 0.67 | 0.0018 |
| All | TiME 8 | PC 1 | Spearman | -0.61 | 0.0057 |
| All | TiME 8 | TiME 9 | Spearman | 0.60 | 0.0068 |
| All | TiME 8 | CD81 | Wilcoxon | 1.99 | 0.0463 |
| All | TiME 9 | TiME 2 | Spearman | -0.64 | 0.0030 |
| All | TiME 9 | TiME 8 | Spearman | 0.60 | 0.0068 |
| All | TiME 9 | Karyotype | Wilcoxon | -2.57 | 0.0102 |
| All | TiME 9 | Type of Bone Marrow | Wilcoxon | 2.56 | 0.0105 |
| All | TiME 9 | Plasma cell percentage in Right CORE | Spearman | -0.57 | 0.0106 |
| All | TiME 9 | TiME 7 | Spearman | 0.55 | 0.0149 |
| All | TiME 9 | PC 1 | Spearman | -0.54 | 0.0163 |
| All | TiME 9 | TiME 3 | Spearman | 0.54 | 0.0182 |
| All | TiME 9 | TiME 11 | Spearman | -0.51 | 0.0249 |
| All | TiME 9 | CD81 | Wilcoxon | 2.13 | 0.0335 |
| All | TiME 9 | FLOW Aberrant plasma cells from total analyzed (%) | Spearman | -0.47 | 0.0435 |
| All | TiME 9 | TiME 12 | Spearman | 0.46 | 0.0461 |
| Overt | PC 0 | cyKAPPA | Wilcoxon | -2.45 | 0.0143 |
| Overt | PC 3 | CD117 | Wilcoxon | 2.07 | 0.0389 |
| Overt | PC 6 | Serum Immunofixation: light chain | Wilcoxon | 2.32 | 0.0201 |
| Overt | PC 7 | CD117 | Wilcoxon | -2.32 | 0.0201 |
| Overt | TiME 0 | CD117 | Wilcoxon | 2.32 | 0.0201 |
| Overt | TiME 11 | cyKAPPA | Wilcoxon | -2.45 | 0.0143 |
| Overt | TiME 11 | CD38 | Wilcoxon | -2.05 | 0.0404 |
| Overt | TiME 2 | CD117 | Wilcoxon | -2.32 | 0.0201 |
| Overt | TiME 5 | CD38 | Wilcoxon | 2.05 | 0.0404 |
| Overt | TiME 6 | cyLAMBDA | Wilcoxon | -2.05 | 0.0404 |
| Precursor | PC 0 | IgM (mg/dL) | Spearman | -0.84 | 0.0024 |
| Precursor | PC 0 | Free Kappa Light Chain (mg/L) | Spearman | 0.70 | 0.0245 |
| Precursor | PC 0 | CD27 | Wilcoxon | -2.17 | 0.0304 |
| Precursor | PC 0 | CD138 | Wilcoxon | -2.09 | 0.0367 |
| Precursor | PC 0 | CD38 | Wilcoxon | -2.09 | 0.0367 |
| Precursor | PC 0 | CD56 | Wilcoxon Rank Sum | -2.09 | 0.0367 |
| Precursor | PC 0 | CD81 | Wilcoxon | -2.09 | 0.0367 |
| Precursor | PC 0 | TiME 3 | Spearman | -0.66 | 0.0368 |
| Precursor | PC 0 | TiME 4 | Spearman | -0.64 | 0.0470 |
| Precursor | PC 2 | Free Kappa Light Chain (mg/L) | Spearman | 0.79 | 0.0063 |
| Precursor | PC 2 | Serum Free Light Chain Ratio (i:U) | Spearman | 0.70 | 0.0233 |
| Precursor | PC 2 | TiME 9 | Spearman | -0.66 | 0.0396 |
| Precursor | PC 4 | TiME 8 | Spearman | 0.68 | 0.0319 |
| Precursor | PC 4 | IgG (mg/dL) | Spearman | 0.66 | 0.0396 |
| Precursor | PC 6 | TiME 11 | Spearman | -0.83 | 0.0032 |
| Precursor | PC 6 | TiME 3 | Spearman | 0.65 | 0.0425 |
| Precursor | PC 7 | Free Kappa Light Chain (mg/L) | Spearman | 0.68 | 0.0294 |
| Precursor | PC 8 | CD117 | Wilcoxon | -2.13 | 0.0330 |
| Precursor | PC 8 | TiME 3 | Spearman | -0.66 | 0.0392 |
| Precursor | PC 8 | CD27 | Wilcoxon | -2.05 | 0.0402 |
| Precursor | PC 8 | cyKAPPA | Wilcoxon | -2.03 | 0.0428 |
| Precursor | PC 9 | TiME 3 | Spearman | 0.65 | 0.0424 |
| Precursor | PC 9 | TiME 8 | Spearman | 0.63 | 0.0491 |
| Precursor | TiME 0 | TiME 1 | Spearman | -0.83 | 0.0029 |
| Precursor | TiME 1 | TiME 6 | Spearman | 0.84 | 0.0022 |
| Precursor | TiME 1 | TiME 0 | Spearman | -0.83 | 0.0029 |
| Precursor | TiME 10 | TiME 8 | Spearman | 0.79 | 0.0061 |
| Precursor | TiME 10 | IgG (mg/dL) | Spearman | 0.73 | 0.0158 |
| Precursor | TiME 10 | CD27 | Wilcoxon | -2.39 | 0.0167 |
| Precursor | TiME 11 | cyKAPPA | Wilcoxon | -2.13 | 0.0330 |
| Precursor | TiME 11 | cyLAMBDA | Wilcoxon | 2.09 | 0.0367 |
| Precursor | TiME 11 | TiME 3 | Spearman | -0.66 | 0.0376 |
| Precursor | TiME 11 | TiME 4 | Spearman | -0.64 | 0.0479 |
| Precursor | TiME 2 | TiME 9 | Spearman | -0.81 | 0.0049 |
| Precursor | TiME 3 | TiME 4 | Spearman | 0.90 | 0.0003 |
| Precursor | TiME 3 | cyKAPPA | Wilcoxon | 2.56 | 0.0105 |
| Precursor | TiME 3 | TiME 11 | Spearman | -0.66 | 0.0376 |
| Precursor | TiME 4 | TiME 3 | Spearman | 0.90 | 0.0003 |
| Precursor | TiME 4 | Free Kappa Light Chain (mg/L) | Spearman | -0.70 | 0.0251 |
| Precursor | TiME 4 | cyKAPPA | Wilcoxon | 2.13 | 0.0330 |
| Precursor | TiME 4 | TiME 11 | Spearman | -0.64 | 0.0479 |
| Precursor | TiME 5 | TiME 7 | Spearman | 0.64 | 0.0479 |
| Precursor | TiME 6 | TiME 1 | Spearman | 0.84 | 0.0022 |
| Precursor | TiME 6 | Free Lambda Light Chain (mg/L) | Spearman | -0.75 | 0.0133 |
| Precursor | TiME 6 | CD45 | Wilcoxon | -2.09 | 0.0367 |
| Precursor | TiME 6 | Karyotype | Wilcoxon | -2.09 | 0.0367 |
| Precursor | TiME 7 | TiME 5 | Spearman | 0.64 | 0.0479 |
| Precursor | TiME 8 | TiME 10 | Spearman | 0.79 | 0.0061 |
| Precursor | TiME 8 | IgG (mg/dL) | Spearman | 0.76 | 0.0111 |
| Precursor | TiME 9 | TiME 2 | Spearman | -0.81 | 0.0049 |
| Precursor | TiME 9 | CD45 | Wilcoxon | -2.09 | 0.0367 |
| Precursor | TiME 9 | Karyotype | Wilcoxon | -2.09 | 0.0367 |
| Precursor | TiME 9 | Type of Bone Marrow | Wilcoxon | 2.09 | 0.0367 |

**Supplemental Figure 1: Survival analysis of cellular phenotypes identified in a-d) NDMM and e-f) overt disease states.** Kaplan-Meier (KM) curve showing that patients with cells from specific phenotypic clusters above or below a specific threshold (median or quartile) had longer overall survival (OS).


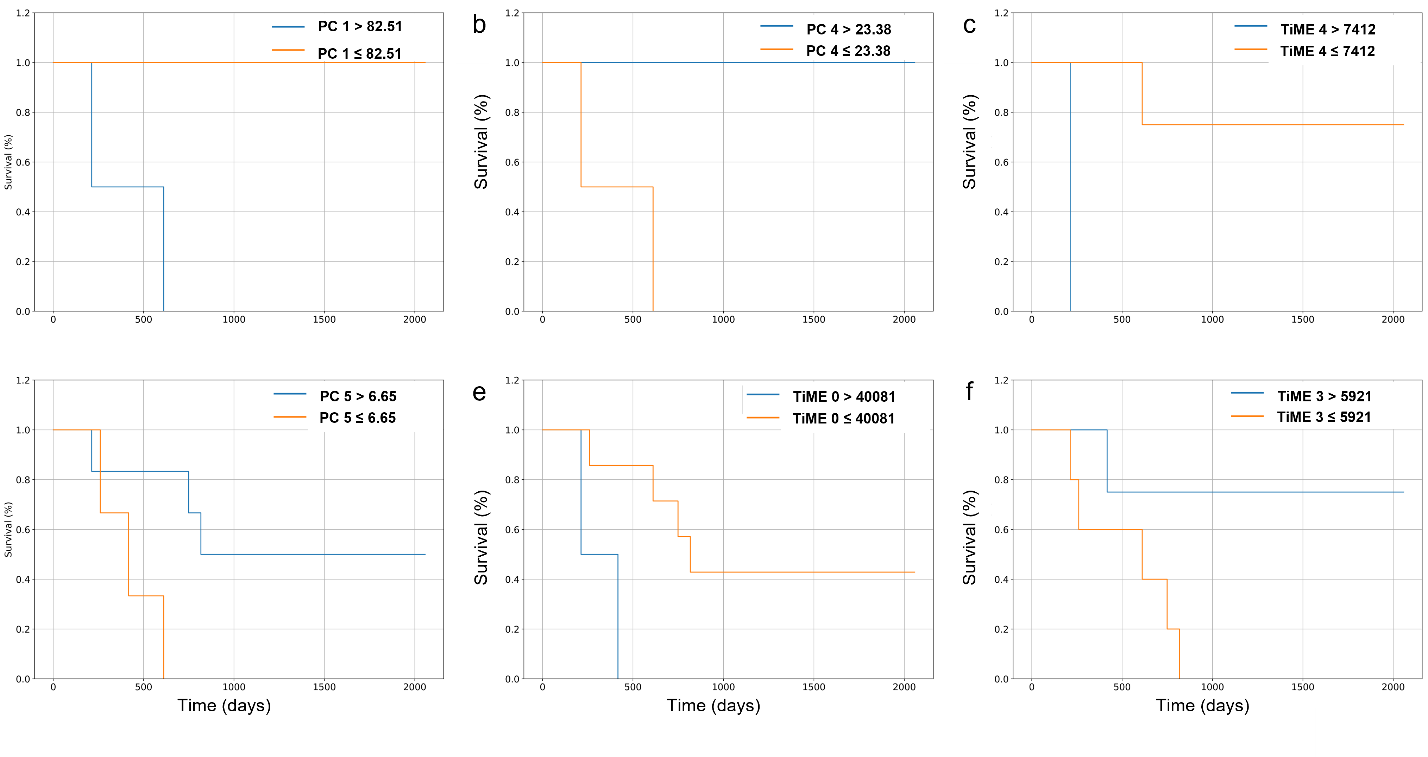

Supplement: Supplementary file 1 [file mmc1.docx]
